# Systematic Review of Chinese Medicine for Miscarriage during Early Pregnancy

**DOI:** 10.1155/2014/753856

**Published:** 2014-02-05

**Authors:** Lu Li, Ping Chung Leung, Tony Kwok Hung Chung, Chi Chiu Wang

**Affiliations:** ^1^Department of Obstetrics and Gynaecology, The Chinese University of Hong Kong, Shatin, New Territories, Hong Kong; ^2^Institute of Chinese Medicine, The Chinese University of Hong Kong, Shatin, New Territories, Hong Kong

## Abstract

*Background*. Miscarriage is a very common complication during early pregnancy. So far, clinical therapies have limitation in preventing the early pregnancy loss. Chinese Medicine, regarded as gentle, effective, and safe, has become popular and common as a complementary and alternative treatment for miscarriages. However, the evidence to support its therapeutic efficacy and safety is still very limited. *Objectives and Methods*. To summarize the clinical application of Chinese Medicine for pregnancy and provide scientific evidence on the efficacy and safety of Chinese medicines for miscarriage, we located all the relevant pieces of literature on the clinical applications of Chinese Medicine for miscarriage and worked out this systematic review. *Results*. 339,792 pieces of literature were identified, but no placebo was included and only few studies were selected for systematic review and conducted for meta-analysis. A combination of Chinese medicines and Western medicines was more effective than Chinese medicines alone. No specific safety problem was reported, but potential adverse events by certain medicines were identified. *Conclusions*. Studies vary considerably in design, interventions, and outcome measures; therefore conclusive results remain elusive. Large scales of randomized controlled trials and more scientific evidences are still necessary to confirm the efficacy and safety of Chinese medicines during early pregnancy.

## 1. Introduction

Miscarriage is defined as spontaneous abortion without medical or mechanical means to terminate a pregnancy before the fetus is sufficiently developed to survive [1]. It denotes early pregnancy loss prior to completion of the 20th gestational week, or 139 days, counting from the first day of the last normal menses [2]. The incidence of miscarriage is commonly stated as 10%–15% of all pregnancies, and it is the most common complication during pregnancy [3]. However, the incidence is difficult to determine precisely, since as many as 30% may go unrecognized, and these can occur very early during a pregnancy. The etiology of miscarriage is largely unknown and the underlying cause of most cases cannot be identified.

Miscarriage can be classified as threatened, inevitable, incomplete, missed, or recurrent. Threatened miscarriage presents as vaginal bleeding/spotting with or without cervical dilatation [1]. It will become inevitable when gross rupture of fetal membranes occurs along with severe vaginal bleeding and cervical dilatation; imminent fetal loss is almost certain in these cases [1]. Incomplete miscarriage refers to the internal cervical os remaining open and allows for passage of blood, but the products of conception could remain entirely or partially in utero extrude [1]. Missed miscarriage is used to describe dead fetus and placenta that remained for days or weeks in the uterus with a closed cervical os and/or without any symptoms of abortion [4]. Recurrent miscarriage is generally defined as spontaneous abortions repeated consecutively over three or more times [1].

Current treatment for miscarriage is rather empirical. Bed rest does not alter the course and progress of miscarriage significantly [5]. Acetaminophen-based analgesia may have some effects on relieving the pains only [6]. Most commonly used Western medicines were progesterone and human chorionic gonadotropin (HCG). HCG maintains the luteotrophic effects after luteinizing hormone secretion decreases in order to support continued secretion of estrogen and progesterone. Progesterone maintains the endometrial proliferation and prevents pregnancy loss [7, 8]. However, their beneficial effect still cannot be verified [7, 9].

The mission of the National Center for Complementary and Alternative Medicine (NCCAM) defines complementary and alternative medicine as a group of diverse medical and health care systems, practices, and products, which are not generally considered part of the conventional medicine [10]. There are different types of CAM [11], including natural products (a variety of herbal medicines, vitamins, minerals, etc.), mind and body medicine (deep-breathing exercises, guided imagery, etc.), and manipulative and body-based practices (spinal manipulation, meditation, and yoga). CAM also encompasses movement therapies (a broad range of Eastern and Western movement-based approaches used to promote physical, mental, emotional, and spiritual well-being) such as Feldenkrais method, Alexander technique, and Pilates.

Chinese medicine is well accepted as the mainstream of medical care throughout East Asia with a history of 5,000 years; it has been spread aboard since the sixth century BC [12]. It has been widely used to promote health and treat illnesses since then [13] and accepted as a major approach of complementary and alternative medicine in Western world now [14]. Chinese medicine includes several different treatments which are applied quite differently, but they are all based on the same understandings of assumptions and insights in the nature of the human body [15]. Main therapeutic approaches [16] include acupuncture (by stimulating certain acu-points with or without acupuncture needles to treat disorders or improve the health condition), Chinese medicines (applying traditional medicines, mainly from herbs, animals, and minerals, to cure illness and maintain good health,), food therapy (dietary recommendations on certain foods and herbs to balance inner body), Qi Gong (promoting health by special breathing and meditation exercise), Tai Chi (benefiting different systems by the movements of muscles and the activities of related joints), Tui Na (applying massage on the surface of the body to clear the meridians and improve the blood flow), Cupping (relieving blood stasis and pain by creating vacuum on body), Die Da (commonly applied in the injuries of limbs by direct stimulation on the body surface), and Gua Sha (scaling the skin to stimulate specific acu-points until mild to moderate subcutaneous hemorrhage) [12, 13, 17].

In Chinese Medicine [18, 19], miscarriage is defined as “fetal irritability” or “fetal restlessness,” while recurrent miscarriage is called “stirring fetus”. Miscarriage shares the same clinical signs and symptoms as in Western Medicine. The presentations of miscarriage are similar, mainly with abdominal pains and vaginal bleeding. But unlike mainstream Western Medicine, Chinese Medicine has a unique medical theory to understand miscarriage. To make the diagnosis and guide the treatment, “Qi” and “Blood” are the two basic elements involved. The major causes of threatened miscarriage include “Kidney Deficiency,” “Qi Deficiency,” “Blood Deficiency,” “Blood Heat,” “External Injury,” and “Wei Jia” (refers to ectopic pregnancy, which is considered as a cause of threatened miscarriage in Chinese Medicine). The diagnosis and treatment are based on different causes and varied a lot in different patients.

The principle [14] of treatments is to supplement and regulate the balance of maternal “Qi,” “Blood,” and the system concerned and enhance the survivals of fetuses, so as to relieve clinical signs, promote pregnancy, and prevent inevitable miscarriage. Besides its application as expectant management for threatened and recurrent miscarriages, Chinese Medicine is also used as active managements for missed, incomplete, and complete miscarriages [20], which mainly accelerate the blood circulation so as to stimulate uterine contractions and empty the uterus.

However, there is only limited information about the application of Chinese medicines for miscarriage in a few systematic reviews recently [21]. Besides, the efficacy and safety claims of Chinese medicines still have no scientific proof. The aim of this study was to access and review the available literature on the clinical applications of Chinese medicine for miscarriage during early pregnancy, in order to provide scientific evidences and valuable references to clinical workers and researchers for practices and studies. The specific objectives were

- to identify the most common therapeutic approach and clinical application of Chinese Medicine for miscarriage;
- to evaluate the most commonly used formulae and individual Chinese medicines for miscarriage and compared the clinical dose and dosing with the recommendations in Chinese Pharmacopeia;
- to analyze the effectiveness and adverse outcomes of Chinese medicines as treatment for miscarriage.

## 2. Materials and Methods

### 2.1. Search Method

We used subject heading, keyword, and abstract including Chinese Medicine, pregnancy, and miscarriage/abortion and searched all published clinical trials of Chinese Medicine for miscarriage. The following databases were searched: Cochrane Central Register of Controlled Trials, mainly Cochrane Database of Systematic Reviews and Cochrane Database of Abstracts of Reviews of Effects (from 1996 to April 2013); EMBASE (from 1980 to April 2013); Cumulative Index to Nursing and Allied Health Literature (CINAHL) (from 1982 to April 2013); Chinese Biomedical Database (CBM) (from 1978 to April 2013); Medline (and PreMedline) (from 1950 to April 2013); China Journal Net (CJN) (from 1915 to April 2013); China National Knowledge Infrastructure (CNKI) (from 1915 to April 2013); Wiley Inter Science (from 1966 to April 2013); and Wan Fang Database (Chinese Ministry of Science and Technology) (from 1980 to April 2013).

We also screened bibliographies of the selected articles and searched by hand for any internet inaccessible articles. We also explored the searches in the reference parts which were listed in these clinical trials and reports identified.

### 2.2. Systematic Review

#### 2.2.1. Inclusion Criteria

- *Types of Studies*. All clinical studies reporting the applications of Chinese Medicine to any miscarriage during early pregnancy were included. All the titles and abstracts were further reviewed.
- *Participants*. All women, regardless of the age, gestational age, parity, and nationality of the participants, receiving Chinese Medicine for miscarriage are included.
- *Diagnosis*. All the studies included for meta-analysis applied the same inclusion criteria—the standard diagnosis and exclusion criteria for threatened miscarriage according to the textbook “Obstetrics & Gynaecology” [18]. Most of the patients received treatment in early pregnancy, around 3rd month of gestation. And there is no significant difference in the baseline of studies. However, as the treatment was individually applied based on the presentation of patients and the experience of Chinese Medicine practitioners, the durations of treatments varied a lot in the included clinical trials, and it is hard to make a comparison or carry out a baseline analysis on this issue.
- *Interventions*. Chinese Medicine was administered as interventions in the clinical trials. Only Chinese medicines recorded by the Chinese Pharmacopeia with well-characterized principles of pharmacological and medicinal applications were included. Other pharmanutrients from various herbal agents and products were excluded. Since Chinese medicines are crude drugs of plant, animal, and mineral origins, not only those Chinese medicines originated from plants or herbs but also those from animals and minerals were included. All types of Chinese medicines in either standard or combined formulas are used in the treatment of threatened miscarriage regardless of the dose or duration of administration.
- *Outcome Measures*. The therapeutic approach and clinical application of Chinese Medicine for miscarriage; the commonly used formulae and individual Chinese medicines and the clinical dose and dosing; the effectiveness and efficacy of the intervention like live birth rate, premature birth rate, miscarriage rate, and the safety of the intervention like side effects and adverse events were studied.
- *Publications*. No restriction on the languages was applied. Publications without full text but with abstracts only were also included.

#### 2.2.2. Exclusion Criteria

- *Other Participants.* Nonpregnancy related and other gynaecology illness, and complications were excluded.
- *Combined Therapies*. If the intervention combined Chinese medicines with other therapies, the clinical trial was included but analyzed seperately.
- *Other Type of Studies*. Case reports, commentary studies, and review articles were excluded.

#### 2.2.3. Data Extraction and Quantitative Analysis

We designed extraction forms to extract data from the selected publications quantitatively. The numbers of the publication in different databases within each decade were counted. The total numbers of included studies were summarized. The total numbers of excluded papers and the exclusion criteria were presented in flow charts. To identify the common clinical applications of Chinese medicines during pregnancy, the clinical indication of each clinical study was recorded and compared. To identify the commonly used Chinese medicine formulae and individual Chinese medicines, the frequency of each formula or individual medicine used in the clinical studies was calculated. The clinical daily dose and dosing of the formula and individual Chinese medicines and their effective rate were recorded and analyzed.

### 2.3. Meta-Analysis

#### 2.3.1. Effectiveness Study

*Study Inclusion*

- *Types of Studies*. Only randomized controlled clinical trials evaluating the effectiveness of Chinese medicines for the treatment of threatened miscarriage were included. Quasirandomized controlled clinical trials (quasirandom method of allocating participants were used) and cluster-randomized trials (participants are recruited in randomized groups) were also studied.
- *Types of Participants*. All women in the clinical trials had a viable pregnancy complicated with threatened miscarriage, regardless of its underlying causes. No treatment was given before interventions. Fetal viability was assessed by ultrasound to exclude the inevitable, incomplete, or missed miscarriage. Vaginal bleeding after the 20th week of pregnancy and recurrent miscarriage were excluded. We included women regardless of whether the pregnancy was singleton or multiple and irrespective of the maternal age and parity.
- *Types of Interventions*. All types of Chinese medicines in either standard or new formulae for the treatment of threatened miscarriage regardless of the dose or duration of administration were compared with other interventions, including no treatment, bed rest, placebo, and other pharmaceuticals.
- *Types of Outcome Measures*. Effectiveness of intervention was defined as either continuation of pregnancy after 28 weeks of gestation or continuation of pregnancy immediately after treatment.

*Study Exclusion*

- *Types of Studies*. Clinical trials without randomization were excluded from this review, but if the randomization method was not clearly stated or was doubted, we contact the author for confirmation.
- *Types of Interventions*. Other therapeutic approaches of Chinese Medicine were excluded. The intervention combined Chinese medicines and other therapies were also excluded from our study.
- *Types of Outcome Measures*. If the trials concluded that Chinese medicines were effective but no data was shown, we also exclude the studies.

#### 2.3.2. Safety Study

*Additional Literature Search.* Since we assumed that the adverse events of Chinese Medicine are very rare, in addition to the above search strategy, we performed additional literature search. To collect the extrainformation on safety of Chinese medicines, several online national and public resources on World Wide Web were also referred, including Center for Food Safety and Applied Nutrition (CFSAN) from U.S. Food and Drug Administration (FDA, http://www.fda.gov/), National Center for Complementary and Alternative Medicine (NCCAM) from U.S. National Institute of Health (NIH, http://nccam.nih.gov/), Agricultural Research Service (ARS) from U.S. Department of Agriculture (USDA, http://www.ars-grin.gov/duke/), Medical Dictionary for Regulatory Activities (MedDRA) from International Federation of Pharmaceutical Manufacturers and Associations (IFPMA, http://www.ifpma.org/), National Council Against Health Fraud (NCAHF) from a private health agency (http://www.ncahf.org/), Quackwatch from an American nonprofit organization (http://www.quackwatch.com/), HerbMed from Alternative Medicine Foundation (http://www.herbmed.org/), and ConsumerLab from an independent laboratory (http://www.consumerlab.com/), accessibility verified until 15 April 2013 [22].

*Inclusion and Exclusion of Study*

- *Types of Studies*. All published clinical studies that evaluated the safety of Chinese medicines for threatened miscarriage were considered. Studies of Chinese medicines in animal, chemical, and basic researches were excluded. Case reports, commentary articles, and nonsystematic reviews were also excluded. Clinical studies with incomplete records or no evaluation of adverse pregnancy outcome were further excluded. Only case controlled studies with or without randomization were included for meta-analysis. Randomized studies, blinded randomized, quasirandomized, and cluster-randomized, were included. Clinical studies without case controlled, including observational and prospective cohorts, were also included for pooled analysis if there are no or too few case controlled studies for invalid meta-analysis.
- *Types of Outcome Measures*. Adverse pregnancy outcomes in both mothers and fetuses/infants will be recorded. Maternal outcomes included toxicity, side effects, pregnancy loss, and pregnancy complications. Fetal outcomes included perinatal mortality, toxicity, congenital malformations, and other neonatal complications.

#### 2.3.3. Assessment of Risk of Bias in Included Studies

We assessed the risk of bias, including the sequence generation to check for possible selection bias, the allocation concealment to check for possible selection bias, the blinding to check for possible performance bias, the incomplete outcome data to check for possible attrition bias through withdrawals, dropouts, protocol deviations, the selective reporting bias, and other sources of bias from compliance and baseline similarity.

#### 2.3.4. Measures of Treatment Effect

Statistical analysis was performed by using Review Manager Version 5.1 (RevMan 5). We presented results as summary risk ratio with 95% confidence intervals for dichotomous data.

#### 2.3.5. Unit of Analysis Issues

Trials with up to three arms (Chinese medicines alone, and Western medicines alone, combined Chinese and Western medicines) were analyzed. We input the data separately for meta-analysis.

#### 2.3.6. Dealing with Missing Data

For included studies, we noted levels of attrition and explored the impact of included studies with high levels of missing data for the overall assessment of treatment effect by using sensitivity analysis. For all outcomes we carried out analyses on an intention-to-treat basis; we attempted to include all participants randomized to each group in the analyses. The denominator for each outcome in each trial was the total number of participants randomized minus any participants whose outcomes were known to be missing.

#### 2.3.7. Assessment of Heterogeneity

We assessed statistical heterogeneity in each meta-analysis using the *T* ^2^, *I* ^2^, and *χ* ^2^ statistics. We regarded heterogeneity as substantial if *T* ^2^ was greater than zero and either *I* ^2^ was greater than 30% or there was a low *P* value (<0.01) in the *χ* ^2^ test for heterogeneity.

#### 2.3.8. Data Synthesis

We carried out statistical analysis using RevMan5 and used fixed-effect inverse variance meta-analysis for combining data when the studies were estimating the same underlying treatment effect and the populations and methods of the trials were judged sufficiently similar. For clinical heterogeneity sufficient to expect that the underlying treatment effects differ between trials, or if substantial statistical heterogeneity was detected, we used random-effects meta-analysis to produce an overall summary if an average treatment effect across trials was considered clinically meaningful. We treated the random-effects summary as the average range of possible treatment effects and we discussed the clinical implications of treatment effects differing between trials. For the average treatment effect which is not clinically meaningful, we did not combine trials. For random-effects analyses, we presented the results as the average treatment effect with its 95% confidence interval and the estimates of *T* ^2^ and *I* ^2^.

For dichotomous safety outcomes, we counted the number of adverse events and the involved participants in each study. For continuous safety outcomes, we calculated the mean and standard deviation of the measures if appropriate. Dichotomous data were expressed as relative ratio (RR) with 95% confidence intervals (CI), while continuous data were expressed as weighted mean differences (WMD) by the meta-analysis.

#### 2.3.9. Subgroup Analysis and Investigation of Heterogeneity

To identify the potential factors of the efficiency outcomes, we also carried out subgroup analyses on maternal age, parity, different trimesters, standard and nonstandard herbal medicines, treatment duration, and quality of included trials. For fixed-effect meta-analyses, we conducted planned subgroup analyses classifying whole trials by interaction tests as described [23]. For random-effects meta-analyses we assessed differences between subgroups by inspection of the subgroups' confidence intervals; non-overlapping confidence intervals indicated a statistically significant difference in treatment effect between the subgroups.

#### 2.3.10. Sensitivity Analysis

We also carried out sensitivity analysis to explore the effect of trial quality for important outcomes in the review. Sensitivity analyses on results were performed on look at the possible contribution of high risk of bias in the allocation of participants to groups associated with a particular study [24] or high levels of missing data [21].

## 3. Results and Discussion

### 3.1. Chinese Medicine for Pregnancy

**Figure 1:**
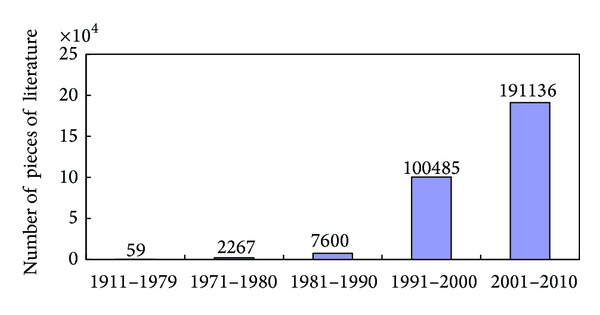
The pieces of literature of Chinese Medicine for all medical fields published in decades. Numbers on the top: total numbers of literature in each decade.

**Figure 2:**
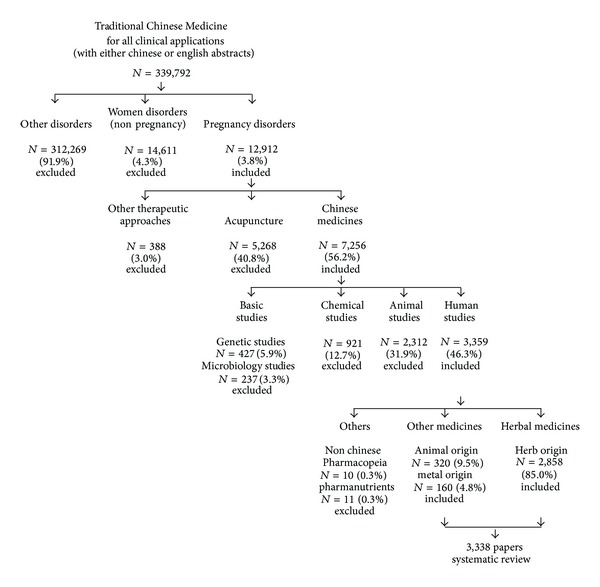
Study inclusions and exclusions for systematic review (Chinese medicines for pregnancy).

**Figure 3:**
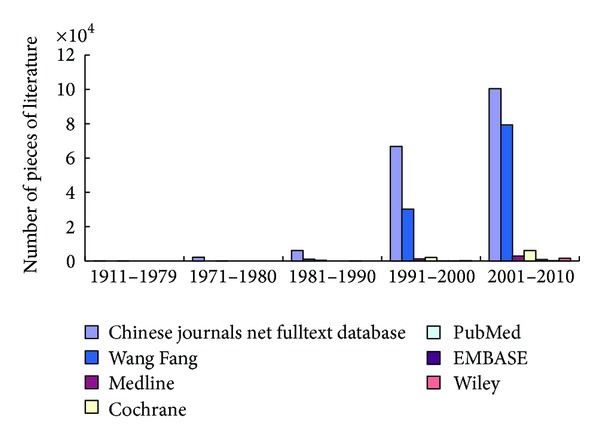
The pieces of literatures of Chinese Medicine for pregnancy published in various databases.

Up to 15 April 2013, 339,792 literature reported studies of Chinese Medicine for all applications were identified (Figure 1). Amongst all the studies, only 12,912 (3.8%) literature studied Chinese Medicine for pregnancy or pregnancy related applications (Table 1). Chinese medicines for other disorders for nonpregnancy applications (91.9%) were excluded (Figure 2). Most of the pieces of literature were mainly found in CNKI and CJN Full-Text Database and WanFang Database but much less in PubMed, Cochrane Library, EMBASE, MEDLINE, and WILEY Interscience (Figure 3). Most of the pieces of literatures (78.6%) were published in Chinese, whilst few of the pieces of literature (21.4%) were published in English and other languages.

In China, it is considered not only as a primary medicine for treatment but also as a supplementary therapy to promote health in general population. Chinese medicines (56.2%) were the most common therapeutic approach of Chinese Medicine for pregnancy, while acupuncture (40.8%) was the second. Amongst all the pieces of literature studied Chinese medicines for pregnancy, human studies (46.3%) were included, while animal studies (31.9%), chemical studies (12.7%), genetic studies (5.9%), and microbiology studies (3.3%) were excluded. Most Chinese medicines are derived from nature, including plants, animals, and minerals. Herbal medicines from plants are much more commonly applied than the others. 2,858 (85.0%) studies used herbal medicines for intervention were included. Other studies used medicines originated from animals (9.5%) and minerals (4.8%) were also included. Other medicines not included by the Chinese Pharmacopeia (0.3%) and included the pharmanutrients from various herbal biological agents and products (0.3%) were further excluded. In total, 3,338 pieces of literature were included for systematic review (Figure 3).

### 3.2. Chinese Medicines for Miscarriage

**Figure 4:**
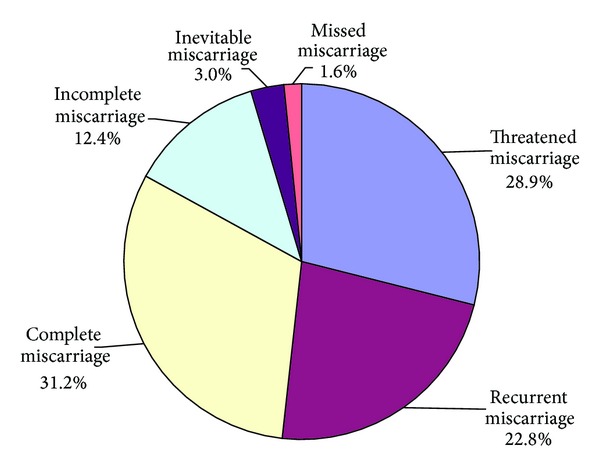
Clinical application of Chinese medicines for different types of miscarriage.

Amongst all pieces of literature of Chinese medicines for pregnancy, miscarriage (43.3%) was the most common clinical indication of the Chinese medicines for pregnancy [25] (Table 2). Less common clinical indications included infertility (27.9%), therapeutic abortion (11.7%), immunological disorders (6.2%), hypertensive disorders (3.8%), infection (2.6%), fetal growth restriction (2.2%), preterm labor (1.5%), postdate (0.3%), gestational diabetes mellitus (0.6%), puerperium (0.1%), and other obstetric complications. Among different types of miscarriage, threatened miscarriage was the most common one for Chinese medicines (Figure 4).

### 3.3. Chinese Medicines for Threatened Miscarriage

**Figure 5:**
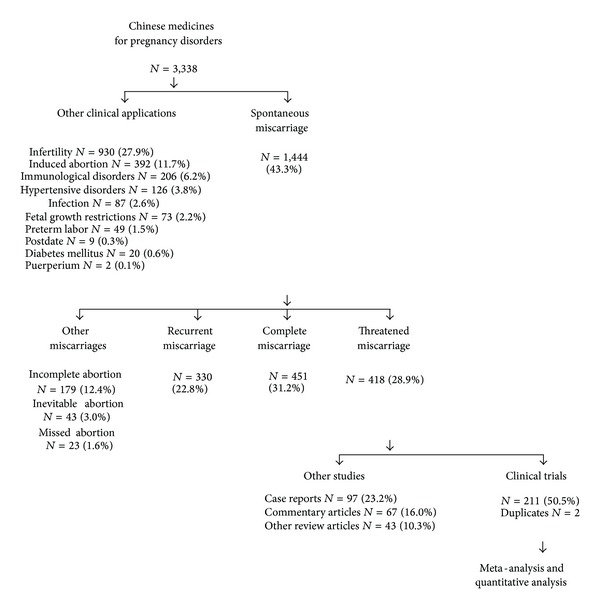
Study inclusions and exclusions for systematic review (e.g., clinical trials of Chinese medicines for threatened miscarriage).

There were 418 clinical studies of Chinese medicines for threatened miscarriages. However, 97 (24.1%) were case reports, 67 (16.0%) were commentary articles, and 43 (10.3%) were review articles. Since case reports are only involved with very small number of participants, mostly with only one case and all less than 5 individuals, which could hardly represent the general application of Chinese medicines. Commentary articles focused on the theory and hypothesis without details and data for further study. Other review articles contributed to summary and conclusion on clinical topics, other than systematically review the clinical trials. We further excluded the case reports, commentary articles, and the review articles. In total, 211 clinical trials 2 duplicated papers excluded, were selected for meta-analysis and quantitative analysis (Figure 5).

#### 3.3.1. Common Formulae

Amongst all the formulae studied for threatened miscarriage (Table 3), “Shou Tai Pill” was the most frequently used formula (72.0%). Its main function is to enhance the function of “Kidney” and regulate the “Qi” in the human body, and then to improve the health condition of mothers and benefit the fetus. Its basic formula includes four individual Chinese medicines, Chinese Dodder Seed (*Semen Cuscuta*), Chinese Taxillus Twig (*Floralia Taxillus*), Himalayan Teasel Root (*Radix Dipsaci*), and Donkey-hide Glue (*Colla Corii Asini*). The former three medicines improve the “Qi” in the body, while Donkey-hide Glue regulates the “Blood” circulation. Therefore, the therapeutic effects of “Shou Tai Pill” are mainly for the pregnant women to improve body condition of mothers and the fetuses. Supplementary of Largehead Atractylodes Rhizome (*Rhizoma Atractylodis Macrocephalae*) and Pilose Asiabell Root (*Radix Codonopsis*) in the formula can enhance the therapeutic effects by further improving the “Qi” of pregnant women [26].

Other popular formulae (Table 3) include “Si Jun Zi Decoction,” “An Tai Yin,” “Wu Zi Decoction,” “An Dian Er Tian Decoction,” and “Jiao Ai Decoction”. The therapeutic effects of “Si Jun Zi Decoction” in the treatment of miscarriage improve the functions of “Spleen” and “Stomach” and regulate “Qi” [27]. “An Tai Yin” improves the function of “Kidney” and then regulates “Qi” and “Blood” [28]. “Wu Zi Decoction” promotes the luteal function by supplementing the “Qi” and “Blood” [29]. “An Dian Er Tian Decoction” relieves the clinical signs such as vaginal bleeding and is mainly applied for recurrent miscarriage [30]. “Jiao Ai Decoction” enhances “Kidney” and regulates “Blood” to improve the health condition of mothers and also to relieve vaginal bleeding [31]. Most of these regimes contain Largehead Atractylodes Rhizome (*Rhizoma Atractylodis Macrocephalae*), Ginseng (*Panax Giaseng*), and Baical Skullcap Root (*Scutellaria Baicalensis*), which are mostly used to nourish and regulate the “Qi”. It has been reported that the combination of Largehead Atractylodes Rhizome and Baical Skullcap Root is highly recommended for their effects to benefit and survive the fetus [17].

In the literature, there were lots of modified formulae, which were not recorded in either Chinese Pharmacopeia or the textbooks. They were prescribed according to the experience of physicians and clinical presentations of the patients. For example, Rehmannia root (*Radix Rehmanniae*), Lycium fruit (*Fructus Lycii*), and Cuscuta fruit (*Semen Cuscutae*) are added into the basic formula of Shou Tai pill and prescribed as a new formula, which was used to correct “Kidney” deficiency in treatment of miscarriage [32].

#### 3.3.2. Individual Chinese Medicines

According to Chinese Pharmacopeia, the official Pharmacopeia for Chinese medicines, acknowledged by World Health Organization (WHO), only 130 out of 1,150 individual Chinese medicines had been used in the clinical trials [29] (see full list in Supplementary Table 1 available online at http://dx.doi.org/10.1155/2014/753856, top 10 list in Table 4).

#### 3.3.3. Clinical Dose and Dosing

A large range of clinical doses of the Chinese medicines was recorded in the reported clinical trials (Supplementary Matrial Table 1). The mean and median daily dose for each medicine ranged from 6 g/day to 23 g/day. About 90.9% of Chinese medicines, 10 to 20 g/day, was recommended, while 9.1% less than 5 g/day or more than 25 g/day was recommended. For example, Fructus Amomi has been prescribed as small as 2 g/day only, while Rehmannia Root has been used more than 30 g/day. In 95% of cases, the Chinese formulae were taken once a day, while 4% twice a day and only 1% three times a day. The top 10 most commonly used single Chinese medicines included Largehead Atractylodes Rhizome, Chinese Dodder Seed, Himalayan Teasel Root, Donkey-hide Glue, Chinese Taxillus Twig, Mongolian Milkcetch Root, White Paeony Root, Chinese Angelica, Liquorice Root, and Baical Skullcap Root in descending order (Table 4).

### 3.4. Efficacy and Effectiveness

#### 3.4.1. Efficacy

**Figure 6:**
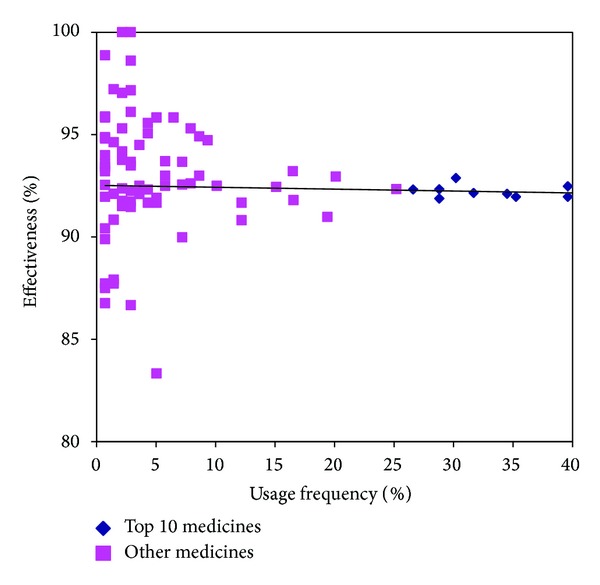
Efficacy of individual Chinese medicines.

Effective rate of Chinese medicines for threatened miscarriages in each clinical trial was recorded [29], and the rate ranged from 75% to 100%. Among all the records, over 84.6% of the studies exceeded 90%, while 24.3% exceeded 95%. Two studies reported 100% [33, 34]. However, no significant correlation was found between frequency of use and effective rate of the studied Chinese Medicine (*r* = 0.1086, *P* = 0.335). The top 10 Chinese medicines only had effective rates from 91.3% to 93.2% (Figure 6). It suggests that commonly used Chinese medicines might not result in a better efficacy.

**Figure 7:**
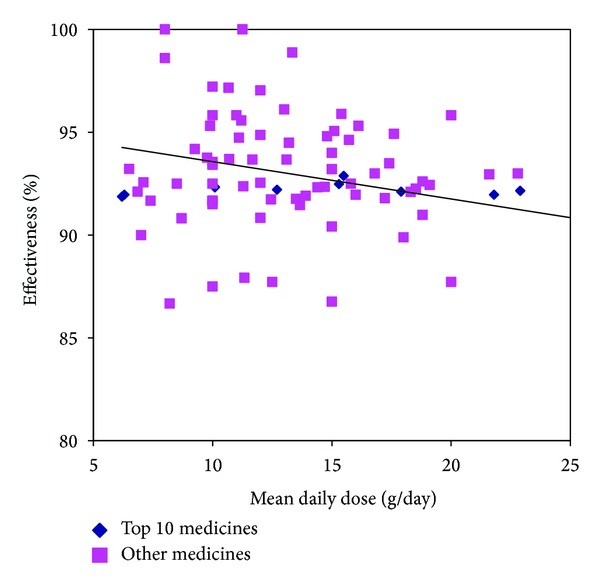
Dosage and efficacy.

To evaluate the relationship between dosage and efficacy of Chinese Medicines in the treatment of threatened miscarriage, the daily dose of the studied Chinese medicine was correlated with the effective rate of the intervention. Chinese medicines with 95% or higher efficacy were less commonly used, 0.72%–7.91%. The Chinese medicines in 20 g or higher mean daily dose, the efficacy ranged from 83.33% to 95.83%. However, there was still no significant correlation between mean daily dose and efficacy (*r* = 0.2324, *P* = 0.513) (Figure 7). It suggests that increased dose might not result in a better efficacy.

**Figure 8:**
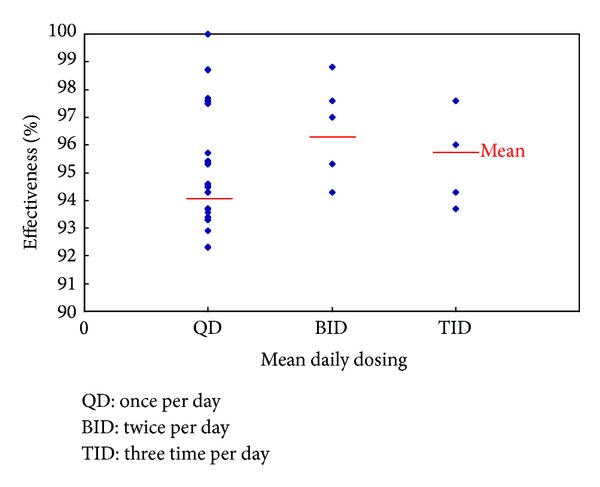
Dosing and efficacy.

There were also different dosing records in different clinical studies. The effective rate of dosing once per day has lowest effective rate, while dosing twice per day has highest effective rate, but dosing three times per day did not further increase the effective rate. There was no significant difference between effective rate and the daily dosing times (Figure 8). It suggests that increasing dosing could not increase the efficacy.

#### 3.4.2. Effectiveness

With a long history of application of Chinese medicines to treat pregnant disorders, large amounts of case reports and clinical trials have been reported [35]. However, until now, no data are available to overview the effectiveness of Chinese medicines for pregnancy.

**Figure 9:**
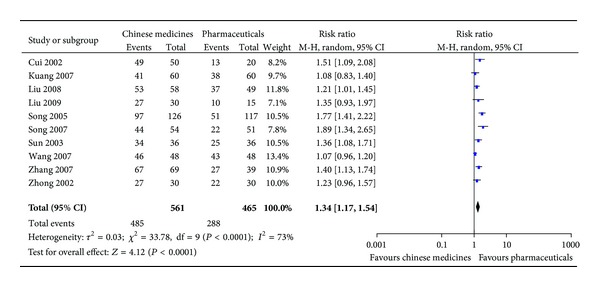
Efficacy of Chinese medicines versus pharmaceuticals.

In our previous review [36], no placebocontrolled trial was found. The effectiveness of Chinese medicine treatments can be compared only through the comparisons amongst Chinese medicines, Western medicines, and combined medicines. However, most Western medicines included tocolytic drugs (e.g., salbutamol and magnesium sulfate), hormonal supplementations (e.g., human chorionic gonadotrophin and progesterone), immunotherapy (e.g., IgG immunization and antiphospholipid antibodies) and supportive supplements (e.g., vitamin E and folic acid), which have no proved benefits. The meta-analysis in limited randomized clinical trials did not support that Chinese medicines alone were more effective than Western medicines. But it showed that combined Chinese and Western medicines were more effective than Western medicines alone to prevent inevitable miscarriage and continue pregnancy after 28 weeks of gestation. Meta-analysis in other clinical trials indicated that Chinese medicines alone or Chinese medicines combined with Western medicines were more effective than Western medicines alone to treat threatened miscarriage in relieving the clinical signs, including vaginal bleeding, low back pain, and abdominal pains. The result confirmed the therapeutic effects of Chinese medicines alone and combined with other pharmaceuticals for threatened miscarriage. In current update study, only one new randomized clinical trial was included [37] for meta-analysis; the new analysis resulted in same conclusion (Figure 9).

Due to insufficiency of data, analysis for all designed subgroups was not employed. Mean maternal age and/or range in each group were not reported. Comparison between the participants of below 35 years old and above 35 years old cannot be performed. All the clinical trials reported the overall parity of all participants but did not provide details about the parity in each groups, so further comparisons were not possible. Gestational age at threatened miscarriage was not available either. Comparison between first trimester and second trimester could not be carried out due to insufficient information. All of the Chinese medicines and the supplements were standard formulae as stated in the Chinese Phamacopeia, so no subgroup analysis between standard formula and nonrecorded formula was carried out. As to the treatment course, only one study [38] reported the termination of intervention (with unknown reason), while the other three studies [39–41] did not report the details on the total amount of the courses for the treatment. Therefore, it is difficult to extract the data and carry out analysis on the duration of intervention for the effectiveness. No trials had any high risk of bias in the allocation of participants to groups or significanty of missing data was identified, so the sensitivity analysis was not carried out. If a sufficient number of trials are found in the future update, we will further specify sensitivity analyses.

Though the result favored Chinese medicines for threatened miscarriage, it must be mentioned that different Chinese medicines were used in different studies. Most Chinese Medicine practitioners slightly modify the standard prescriptions depending on the presentations of each patient. In the clinical trials, most of the trials used a common prescription of “Shou Tai Pill” as basic formula, while the others used different prescriptions. However, some Chinese medicines have been supplemented into or removed from the standard formula during the treatment. Therefore, the effectiveness of the studies could only represent the general effects of Chinese medicines but not the effects of the Chinese formula or individual Chinese medicines. Besides, the success rate for many of the clinical trials was higher than that in the general population (often over 90%). This indicated that many of the patients included were likely relatively far along in the pregnancy (when loss rates are lower).

As in most cases, doctors would suggest the patients to have bed rest first, although it also has no significant effects in altering the course and progress of miscarriage [5]. However, no study compared Chinese medicines treatment to bed rest in this review. Western medicines were not considered as classical therapies for threatened miscarriage [7, 8]. So the conclusion of the effectiveness of Chinese medicines is limited. Therefore, more placebocontrolled trials are necessary to evaluate the effectiveness of Chinese medicines.

#### 3.4.3. Limitations and Difficulties

In the early years of Chinese Medicine studies, the pieces of literature could be only obtained from Chinese databases. With the development of Chinese Medicine and its spread to foreign countries, more and more western scientists and clinical workers have interests in Chinese Medicine, and various studies have been carried out and could be identified in English databases since the late 70s. An increasing trend is that Chinese Medicine was studied by foreign researchers in the following decades. From 2000 onwards, more English database recorded Chinese Medicine in different areas and topics, covering clinical trials for various applications, animal studies for toxicity tests, laboratory research on chemical components, and commentary articles for theories of Chinese Medicine. Due to the differences in language and theory, most pieces of literature of Chinese Medicine studies are still identified in Chinese database, however. There are some major limitations in identifying the publications from various databases. As there are some discrepancies in the translation of Chinese Medicine from Chinese to English, and lots of medical terms of Chinese Medicine are difficult to interpret, literature searches are always inefficient when searched by an English subject headings and keywords in Chinese database. On the other hand, 80% of the pieces literature were overlapped in two famous Chinese databases, China National Knowledge Infrastructure (CNKI) and WanFang, and it is time consuming to double check because of limited resources and tools. As the mainstream medicine is in China, over 86% of the publications were found in Chinese databases only. This largely limits the researchers and scientists in western countries to obtain the information and knowledge. Although some of the publications were with English abstracts, in most cases English full texts are not available. It is very difficult for foreigners to understand the Chinese Medicine.

Regarding the design of the clinical studies in Chinese medicines for threatened miscarriage, there are still some limitations. Firstly, well-conducted randomized controlled trials are important for meta-analysis. Most of the selected trials have inadequate methodology quality. Furthermore, the quality of each clinical trial was obviously not at the same level. Therefore, it would be greatly helpful to improve the quality of analysis if the authors were adequately trained to carry out and report such clinical trials according to the international standard, including sufficient randomization method and adequate allocation concealment, double-blinded participants and researchers or outcome assessors, participants' classifications, and effects assessments. Secondly, a better clinical trial should also provide some essential information, such as the average days or weeks of the treatments, the changes in the medicines dosage and compositions, the amount of participants with a successful pregnancy till 28 weeks or afterwards, and the mortality and follow-ups of newborns, which would be helpful to examine the effects of Chinese medicines in the treatment. Thirdly, all the comparisons in these clinical trials were made between Chinese medicines and other medicines, which were not the recommended and the most effective treatment for threatened miscarriage. So it is hard to draw a conclusion on the therapeutic effects of Chinese medicines, and we suggest more placebocontrolled trials to be involved in the future clinical trials. Last but not least, small numbers of qualified clinical trials and insufficient information in this review did limit us to conduct further subgroup analysis; more details and information of the clinical trials are essential to further understand the effects of Chinese medicines in the analysis.

### 3.5. Safety and Adverse Outcomes

#### 3.5.1. Safety

So far, Chinese medicines are claimed to be safe if applied properly, and in the last 3000 years of practices, most of the medicines have been applied in the same way as their first records. In fact, all Chinese Medicine practitioners agree that Chinese medicines are with their potential side effects, which could be reduced or eliminated by prescribing in formula, shortening the course of treatment and correctly adjusting the dose and constitutes of Chinese medicines. However, the scientific evidence of its safety is still lacking.

Despite case reports of adverse effects accused by Chinese medicines, evidences of adverse events in the use of Chinese medicines for threatened miscarriage are limited. In most clinical trials, adverse effects and toxicity of the Chinese medicines were not studied. Only very few studies reported no adverse effect and toxicity after maternal exposure [42–44] and some gastrointestinal effects, including nausea, dry mouth, anorexia, and constipation were reported [43]. Clinical outcomes of threatened miscarriage were not followed in about 2%–20% cases, if Chinese medicines intervention failed. Even if the intervention was successful and pregnancies were maintained, adverse pregnancy and/or perinatal outcomes were not studied in most studies. Preterm deliveries were reported in some studies with incidence rate 0.7%–6.4% [45–48], while premature rupture of membranes and stillbirth were identified in a separate study [46]. Neonatal death due to prematurity, asphyxia, or infection was identified in some studies [45–47, 49], while an unspecified malformation was recorded in a study [35] and epilepsy and mental retardation in a study [47]. However, all the clinical studies did not further study and explain the possible causes for these adverse effects, and there was limited evidence for us to find a direct relation between the adverse outcomes with Chinese medicines.

#### 3.5.2. Adverse Outcomes

**Figure 10:**
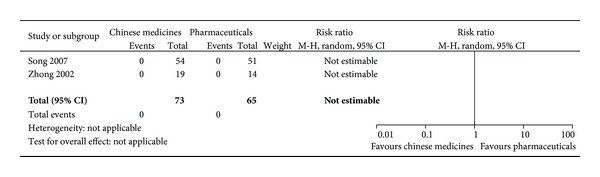
Adverse outcome of Chinese medicines versus pharmaceuticals.

In our previous review [21], thirty-two relevant articles included 9 randomized controlled trials, 1 quasirandomized controlled trial, and 2 controlled trials comparing Chinese medicines alone or combined medicines with pharmaceuticals and 20 case series with no controls were studied and analyzed. However, sample sizes of each study were generally small. There was variation in Chinese medicine formulation, dosage, and duration of treatment. In the pooled randomized controlled trials, dry mouth, constipation and insomnia (2–10%), intervention failure (3.1–22.3%), diabetic complications (3%), preterm delivery (5%), and neurodevelopmental morbidity (1.8%) were recorded. Meta-analysis demonstrated that intervention failure was significantly lower in the combined Chinese medicines groups than in the Western medicines controls (relative risk = 0.46; 95% confidence interval: 0.30–0.70, I^2^ = 0%). No significant differences were found between these groups for adverse effects and toxicity or for adverse pregnancy and perinatal outcomes (Figure 10). In current update study, no new randomized clinical trial was included for meta-analysis.

Chinese medicines are not free of risk; similar to Western pharmaceutical medicines, they have the potential to cause adverse effects. The active ingredients of Chinese medicines are also chemicals that are similar to prescription pharmaceuticals. Thus, Chinese medicines in Chinese Medicine may not only result in maternal manifestations that indirectly affect fetal health, but they may also harm the fetus directly. Despite variations in clinical practice and therapeutic prescription, Chinese medication in Chinese Medicine should comply with the same modern pharmacological principles as Western Medicine. Chinese medicines not only may be beneficial but may also adversely affect both mothers and fetuses in utero.

#### 3.5.3. Limitations and Difficulty

Over 90% of the clinical trials did not include adverse effect as one of study outcome measures. It may due to the incidence of adverse events be indeed too rare or the awareness on the adverse effects was actually too low. The low rate of adverse events after maternal exposure to Chinese medicines for threatened miscarriage was recorded, however. The incidences were not too low to be easily missed. Hence lack of awareness on the safety issue of Chinese medicines in general could be the main reason for limited safety studies available.

Apart from limited records in adverse outcomes, study designs were also restricted. There were no placebocontrolled trial and only 1 controlled trial was with adequate randomization method. Whilst all other cohorts had no controls for comparison, allocation methods were not described and their quality was relatively low. Nevertheless, the study results were questionable. Though the drop-out rates were not high, sample sizes of the selected studies were still very small. Some important demographic data and study exclusion criteria were not provided. Different studies used different Chinese medicine formulae to treat threatened miscarriage and also there were large variations in the dose, dosing, and duration of the intervention amongst the studies. Most studies followed up the pregnancy until delivery, but the outcome parameters in the pregnancy and perinatal complications were rather inconsistent. Few studies monitored adverse effects and toxicity of Chinese medicines, and complicated miscarriage was unknown. Owing to the limited number of randomized controlled trials and the clinical heterogeneity between studies, additional meta-analysis to evaluate the adverse effects of Chinese medicine for threatened miscarriage was not available.

Unlike Western herbalism, Chinese medicines include many animal materials and even mineraloid remedies as well as medicinal herbs [50]. Most of the formulae are processed by decoction in boiling water for hours and are orally administered as a “soup” [51], though it can be supplied as powders, soluble granules, and tablets nowadays. As each Chinese medicine has its own property and potential interaction, the application of formulated and individualized medication enhances the therapeutic actions of some herbs and collaborates all the herbs to balance disharmony of each individual for treatment. However, the adverse effects and toxicity of Chinese medicines may vary in different combinations, preparations, and individuals. It is difficult to identify which Chinese medicine attributes to the specific adverse effects.

## 4. Conclusions

Chinese medicines, as the most common therapeutic approach of Chinese Medicine, have become popular for therapeutic and complementary use in healing diseases and maintaining health, not only in China but also around the world. In this study, we focused on the Chinese medicines for pregnancy providing background information for overall understanding on the clinical applications of Chinese Medicine during pregnancy, and scientific evidences on their efficacy and safety for pregnancy use.

The results showed that (1) 339,792 pieces of literature were identified, mostly from Chinese databases, and only few clinical studies were selected for systematic review; (2) Chinese medicine is the most common therapeutic approach for miscarriage, and the most common clinical indication was threatened miscarriage; (3) the most commonly prescribed formula to prevent miscarriage and promote the pregnancy was Shou Tai Pill, and the most frequently used individual Chinese medicine was Largehead Atractylodes Rhizome; (4) the Chinese medicines for threatened miscarriage were mostly single dose per day, but the range of the dose was quite large; (5) the overall effectiveness rate of Chinese medicines for threatened miscarriage was over 90%; however, there is no direct correlation between efficacy and usage frequency, and between efficacy and dose and dosing; (6) no placebo was included in either efficacy or safety study, and only few studies with high quality were conducted for meta-analysis; (7) based on limited clinical trials, a combination of Chinese medicines and Western medicines was more effective than Chinese medicines alone. No specific safety problem was reported, but potential adverse and toxic effects by certain medicines were identified.

It is suggested that Chinese medicines combined with Western medicines may be effective to treat miscarriage and relieve the clinical signs, while Chinese medicines alone may not be effective. However, large scales of randomized controlled trials and more scientific evidences, especially placebocontrolled trials, are still necessary to confirm the effectiveness of Chinese medicines. In most of the pieces of literatures, Chinese medicines are 90% effective, and our meta-analysis also supports the therapeutic application of Chinese medicines. However, most of the studies are so flawed that meaningful conclusions cannot be made; there is a desperate need for better clinical studies on Chinese medicines. Potential adverse effects of Chinese medicines on mothers and fetuses during pregnancies are lacking, and the evidences of adverse events in the use of Chinese medicines for threatened miscarriage are limited. Studies vary considerably in design, interventions, and outcome measures; therefore conclusive results remain elusive. Rigorous scientific and clinical studies are necessary to confirm the risk of Chinese medicines.

## Supplementary Material

The information of Chinese medicines commonly applied as treatments for threatened miscarriage has been list in the Supplementary Table 1, including the Chinese, English and Biological names, the frequency of usage, the recorded dose in “Chinese Pharmacopeia” and the daily dose in all published literatures of each Chinese medicine.

## Figures and Tables

**Table 1:** Number of literature studies on clinical application of Chinese Medicine.

Chinese Medicine approaches	Clinical application in all medical fields	Clinical application for pregnancy	Clinical application for miscarriage
Chinese medicines	241,237 (80.0%)	3,338 (90.5%)	1,444 (91.1%)
Acupuncture	28,754 (9.5%)	208 (6.2%)	96 (6.2%)
Food Therapy	13,382 (4.5%)	96 (2.9%)	32 (2.0%)
Qi Gong	11,671 (3.9%)	7 (0.2%)	2 (0.1%)
Tui Na	3,061 (1.0%)	16 (0.5%)	2 (0.1%)
Tai Chi	1,646 (0.5%)	7 (0.2%)	2 (0.1%)
Cupping	864 (0.3%)	8 (0.2%)	2 (0.1%)
Die Da	529 (0.2%)	8 (0.2%)	2 (0.1%)
Gua Sha	403 (0.1%)	1 (<0.1%)	3 (0.2%)

Total	301,547 (100%)	3,689 (100%)	1,585 (100%)

**Table 2:** Clinical applications of Chinese medicines during pregnancy.

Pregnancy disorders	Frequency* (%)	Therapeutic applications
Spontaneous abortion	1,444 (43.3)	Improve maternal health Promote embryo-fetal development Reduce irregular vaginal bleeding

Infertility	930 (27.9)	Improve women's health Elevate the female fertility

Induced abortion	392 (11.7)	Enhance lethal effect on embryos Decrease incomplete abortion rate Improve vaginal irregular bleeding

Immunological disorders	206 (6.2)	Inhibit the release of inflammatory molecules

Hypertensive disorders	126 (3.8)	Promote vasodilatation Increase blood flow Decrease platelet aggregation

Infection	87 (2.6)	Decrease intrauterine transmission

Fetal growth restriction	73 (2.2)	Improve uteroplacental circulation Promote fetal growth

Preterm labour	49 (1.5)	Inhibit uterine contractility

Postdate or term	9 (0.3)	Accelerate labor process

Gestational diabetes mellitus	20 (0.6)	Improve insulin levels Enhance glucose metabolism

Puerperium	2 (0.1)	Improve hormone levels Promote lactation and uterine contraction Heal perineum injuries

Total	3,338 (100)	

*Frequency of each clinical application amongst 3,228 papers for systemic review.

**Table 3:** Chinese medicine formulae studied for threatened miscarriage.

Order	Name	Frequency*	Main composition	Therapeutic actions			Other applications^b^
				Dose^a^ (g)	Dosing^a^	System/organ	
1	*Shou Tai Pill *	301 (72.0%)	*Colla Corii Asini* *Herba Taxilli* *Radix Dipsaci* S*emen Cuscutae *	5–15 9–15 9–15 6–12	QD/BID	Reproductive system and Spleen and Kidney	Abdomen distension, lower abdomen pain, dizziness, frequent urination, urinary incontinence, tinnitus, lower-limb weakness.

2	*Si Junzi Decoction *	52 (12.5%)	*Ginseng, Poria* *Radix Glycyrrhizae* *Rhizoma Atractylodis Macrocephalae *	9–30 3–9 6–12	QD/BID	Immune system and Kidney and Spleen	Chronic gastritis, gastric ulcer, duodenal ulcer, antitumor

3	*An Tai Decoction *	28 (6.7%)	*Cortex Eucommiae* *Folium Artemisiae argyi* *Giseng,* *Radix Dipsaci Poria cocos* *Radix Angelicae sinensis* *Radix Astragali* *Radix Paeoniae Alba* *Radix Rehmanniae Praeparata,* *Cyperi Radix* *Scutellariae Rhizoma* *Atractylodis Macrocephalae Rhizoma *	6–15 3–9 9–30 9–15 6–12 9–30 6–15 9–15 6–9 9–30 6–12	QD/BID	Reproductive system and Kidney and Spleen	Vitiligo

4	*Wu Zi Decoction *	16 (3.8%)	*Caulis Perillae* *Colla Corii Asini* *Cortex Eucommiae* *Fructus Lycii, Fructus Rubi* *Fructus Schisandrae chinensis,* *Herba Taxilli* *Radix Dipsaci* *Radix Scutellariae* S*emen Cuscutae *	5–9 5–15 6–15 6–12 3–10 3–6 9–15 9–15 9–30 6–12	QD/BID	Kidney	Uterine hypoplasia, male infertility

5	*An dian Er tian Decoction *	10 (2.4%)	*Eucommia ulmoides Oliver* *Fructus Corni, Preparata* *Fructus Lycii,* *Giseng* *Radix Glycyrrhizae* *Radix Rehmanniae* *Rhizoma Atractylodis* *Macrocephalae* *Rhizoma Dioscoreae *	6–15 6–12 6–12 9–30 3–9 9–15 6–12 6–12 15–30	QD/BID	Spleen Kidney	Postmenopausal bleeding

6	*Jiao Ai Decoction *	6 (1.4%)	*Colla Corii Asini* *Folium Artemisiae Argyi* *P. Lactiflora Pall* *Radix Angelicae Sinensis* *Radix Glycyrrhizae* *Radix Rehmanniae Praeparata *	5–15 3–9 6–15 6–12 3–9 9–15	QD/BID	Liver Spleen Kidney	Thrombocytopenic purpura, abdominal pain

7	Others	5 (1.2%)	Nonstandard Formulae	3–30	QD/TID	Spleen	

*% is the number of literature of each formula/total amount of literature × 100.

^a^Therapeutic dose and dosing refer to the dose and dosing of the formulae per regime for threatened miscarriage; QD: once per day; BID: twice per day; TID: three times per day.

^b^Other applications refer to the applications of the formulae for other disorders during pregnancy.

**Table 4:** Top 10 of most commonly studied individual Chinese medicines for threatened miscarriage.

Order	English name	Biological name	Frequency*	Mean daily dose^a^	Therapeutic actions^b^	Other applications^c^
1	Largehead Atractylodes Rhizome	*Rhizoma Atractylodis Macrocephalae *	59 (41%)	12.7 g	Prevent miscarriage	—

2	Chinese Dodder Seed	*Semen Cuscutae *	55 (38%)	21.8 g	Prevent miscarriage and prelabor	Cataract, diarrhea, sperm abnormality, chronic prostatitis.

3	Himalayan Teasel Root	*Radix Dipsaci *	55 (38%)	15.3 g	Stop vaginal bleeding Prevent miscarriage	Fractures and injuries, Lower back pain.

4	Donkey-hide Glue	*Colla Corii Asini *	49 (35%)	6.3 g	Increase platelet count Stop vaginal spotting	Chronic bleeding, anemia, tuberculosis, uterine fibroids, endometriosis.

5	Chinese Taxillus Twig	*Herba Taxilli *	48 (34%)	17.9 g	Prevent miscarriage Lower high blood pressure	Lower back pain, tendons atrophy.

6	Milkvetch Root	*Radix Astragali *	48 (34%)	22.9 g	—	Chronic nephritis, diabetes mellitus, diuresis.

7	White Peony Root	*Radix Paeoniae Alba *	44 (31%)	15.5 g	Regulate menstruation	Abdomen and limb pain, check sweating.

8	Chinese Angelica	*Radix Angelicae Sinensis *	42 (29%)	10.1 g	Improve blood circulation Regulate menstruation	General pain, bowels overactivity.

9	Liquorice Root	*Radix Et Rhizoma Glycyrrhizae *	40 (28%)	6.2 g	—	Detoxification, dispel phlegm, coughing, spasmodic pain.

10	Baical Skullcap root	*Radix Scutellariae *	35 (24%)	10.1 g	Stop vaginal bleeding Prevent miscarriage	Detoxification.

*% is the number of literature of each formula/total amount of literature ∗ 100.

^a^The mean of the reported daily dose in all included clinical trials.

^b^Functions of Chinese herbal medicines as treatment to threatened miscarriages.

^c^Other functions of Chinese herbal medicines as treatment to other disorders.
