# Supplementary material for: Systematic Review of Chinese Medicine for Miscarriage during Early Pregnancy
Source: Evid Based Complement Alternat Med. 2014 Feb 5;2014:753856. doi: 10.1155/2014/753856 (PMC3933529; doi:10.1155/2014/753856)
Supplement: Supplementary file 1 — The information of Chinese medicines commonly applied as treatments for threatened miscarriage has been list in the Supplementary Table 1, including the Chinese, English and Biological names, the frequency of usage, the recorded dose in “Chinese Pharmacopeia” and the daily dose in all published literatures of each Chinese medicine. [file 753856.f1.pdf]

Supplementary Table1. Common Chinese medicines for threatened miscarriage

Order	Chinese names	English names	Biological names	Frequency (%)*	Remmended Dose[#] min-max (g)	Daily Mean Dose[※] ± SD (g)
1	白术	Largehead Atractylodes Rhizome	*Rhizoma Atractylodis Macrocephalae*	59 (41%)	6-12	12.7 ± 4.40
2	菟丝子	Chinese Dodder Seed	*Semen Cuscutae*	55 (38%)	6-12	21.8 ± 8.83
3	续断/川断	Himalayan Teasel Root	*Radix Dipsaci*	55 (38%)	9-15	15.3 ± 5.63
4	阿胶	Donkey-hide Glue	*Colla Corii Asini*	49 (35%)	5-10	6.4 ± 2.02
5	桑寄生	Chinese Taxillus Twig	*Herba Taxilli*	48 (34%)	9-15	17.9 ± 7.81
6	甘草	Liquorice Root	*Glycyrrhizae,Radix Et Rhizoma Glycyrrhizae Praeparata Cum Melle*	48 (34%)	1.5-9	6.2 ± 12.07
7	黄芪	Mongolian Milkcetch Root	*Radix Astragali*	44 (31%)	9-30	22.9 ± 5.50
8	白芍	White Paeony Root	*Radix Paeoniae Alba*	42 (29%)	6-15	15.5 ± 2.58
9	当归	Chinese Angelica	*Radix Angelicae Sinensis Radix Et Rhizoma*	40 (28%)	6-12	10.1 ± 2.67
10	黄芩	Baical Skullcap Root	*Radix Scutellariae*	35 (24%)	9-30	10.1 ± 2.36
11	杜仲/杜仲炭/炒杜仲	Eucommia Bark	*Cortex Eucommiae*	35 (24%)	6-9	14.7 ± 4.47
12	熟地/熟地黄	Steamed Rehmannia Root	*Radix Rehmanniae Praeparata*	28 (20%)	9-15	21.6 ± 2.65
13	党参	Pilose Asiabell Root/ Szechwon Tangshen Root	*Radix Codonopsis*	27 (19%)	9-30	18.8 ± 3.89

14	山药/怀山药	Common Yam Rhizome/ Wingde Yan Rhizome	*Rhizoma Diosscoreae*	23 (16%)	15-30	20.8 ± 2.67
15	砂仁	Villous Amomrum Fruit	*Fructus Amomi*	23 (16%)	3-6	6.5 ± 2.13
16	地黄/生地黄/生地	Rehmannia Root	*Radix Rehmanniae*	22 (15%)	9-15	19.1 ± 2.54
17	川芎	Szechuan Lovage Rhizome	*Rhizoma Chuanxiong*	17 (12%)	3-9	7.4 ± 2.26
18	艾叶/艾叶炭	Chinese Mugwort Leaf	*Folium Artemisiae Argyi*	17 (12%)	3-9	8.7 ± 2.44
19	益母草	Motherwort Herb	*Herba Leonuri*	15 (10%)	10-15	14.5 ± 1.05
20	陈皮	Tangerine Peel	*Pericarpium Citri Reticulatae*	14 (10%)	3-9	8.5 ± 2.93
21	丹参	Danshen Root	*Miltiorrhizae*	14 (10%)	9-15	11.1 ± 7.05
22	太子参	Heterophylly Falsestarwort Root	*Radix Pseudostellariae*	14 (10%)	9-30	17.6 ± 2.77
23	旱莲草	Giant St.John's Wort Herb	*Herba Ecliptae Eclipta prostrala L*	14 (10%)	15-30	16.8 ± 5.10
24	紫苏梗/苏梗/紫苏	Perilla Stem	*Caulis Perillae*	14 (10%)	5-9	9.9 ± 3.40
25	苎麻根	Ramie Root	*Radix Boehmeriae*	14 (10%)	5-30	18.8 ± 5.65
26	人参	Gin Seng	*Radix Ginseng*	11 (8%)	3-10 or 10-30	10.6 ± 10.41
27	茯苓	Indian Buead	*Poria*	10 (7%)	9-15	13.6 ± 5.84
28	升麻/炙升麻	Largetrifoliolious Bugbane Rhizome	*Rhizoma Cimicifugae*	10 (7%)	3-9	7.1 ± 1.52
29	柴胡	Chinese Thorowax Root	*Radix Bupleuri*	10 (7%)	3-9	7 ± 1.14
30	赤芍	Red Paeony Root	*Radix Paeoniae Rubra*	9 (6%)	6-12	11 ± 2.43

31	女贞子	Glossy Privet Fruit	*Fructus Ligustri Lucidi*	8 (6%)	6-12	15.8 ± 8.78
32	仙鹤草	Hairyvein Agrimonia Herb and Bud	*Herba Agrimoniae*	8 (6%)	6-12	22.8 ± 10.24
33	竹茹/姜竹茹	Bamboo Shavings	*Caulis Bambusae in Taenia*	8 (6%)	4.5-9	10.7 ± 4.33
34	山萸肉/山茱萸	Common Macrocarpium Fruit	*Fructus Corni*	7 (5%)	6-12	13.9 ± 2.43
35	蒲黄/生蒲黄/炒蒲黄	Cattail Pollen	*Pollen Typhae*	7 (5%)	5-9	10 ± 3.46
36	糯米	Polished Glutinous Rice	*Semen Oryzae Glutinosae*	7 (5%)	30-60	30 ± 0.00
37	郁金	Turmeric Root Tuber	*Radix Curcumae*	7 (5%)	3-9	10 ± 3.03
38	补骨脂	Malaytea Scurfpea Fruit	*Fructus Psoraleae*	6 (4%)	6-9	14.4 ± 3.45
39	泽兰	Hiraute Shiny Bugleweed Herb	*Herba Lycopi*	6 (4%)	6-12	10 ± 4.64
40	枸杞/杞果	Barbary Wolfberry Fruit	*Fructus Lycii*	6 (4%)	6-12	15.1 ± 8.01
41	香附	Nutgrass Galingale Rhizome	*Rhizoma Cyperi*	6 (4%)	6-9	11.2 ± 7.54
42	鸡血藤	Suberect Spatholobus Stem	*Caulis Spatholobi*	5 (3%)	9-15	18.3 ± 7.09
43	茜草根	India Madder Root	*Radix Et Rhizoma Rubiae*	5 (3%	6-9	13.2 ± 7.66
44	桂枝	Cassia Twig	*Ramulus Cinnamomi*	5 (3%)	3-9	6 ± 0.96
45	鹿角/鹿角胶霜	Deerhorn	*Colla Cornu Cervi ,Cornu Cervi Degelatinatum*	5 (3%)	5-10	10 ± 2.89
46	酸枣仁	Spina Date Seed	*Semen Ziziphi*	5 (3%)	9-15	13.7 ± 8.22

			Spinosae			
47	延胡索/延胡/元胡	Yan Hu Suo	*Rhizoma Corydalis*	5 (3%)	3-9	11 ± 1.89
48	小茴香	Fennel Fruit	*Fructus Foeniculi*	4 (3%)	3-6	3 ± 0.88
49	巴戟/巴戟天	Medicinal Indianmulberry Root	*Radix Morindae Officinalis*	4 (3%)	3-9	12.4 ± 2.50
50	地榆/地榆炭/炒地榆	Garden Burnet Root	*Radix Sanguisorbae*	4 (3%)	9-15	18.5 ± 3.54
51	麦冬	Dwarf Lilyturf Tuber	*Radix Ophiopogonis*	4 (3%)	6-12	13 ± 1.41
52	荆芥/荆芥炭	Fineleaf Schizonepeta Herb	*Herba Schizonepetae (Carbonisatum)*	4 (3%)	5-10	10.7 ± 3.08
53	栀子	Cape Jasmine Fruit	*Fructus Gardeniae*	4 (3%)	6-9	8.2 ± 2.03
54	香橼	Citron Fruit	*Fructus Citri*	4 (3%)	3-6	5 ± 0.35
55	莲房炭	Lotus Seed Pot	*Receptaculum Nelumbinis*	4 (3%)	5-10	5 ± 0.0
56	益智仁	Sharpleaf Galangal Fruit	*Fructus Alpiniae Oxyphyllae*	4 (3%)	3-9	17.4 ± 7.64
57	五灵脂	Trogopterus Dung	*Faeces Trogopterori*	4 (3%)	5-10	8 ± 1.15
58	川楝子	Szechwan Chinaberry Fruit	*Fructus Toosendan*	3 (2%)	4.5-9	9.25 ± 2.58
59	五味子	Chinese Magnoliavine Fruit	*Fructus Schisandrae Chinensis*	3 (2%)	1.5-6	10 ± 1.24
60	半夏/法半夏	Pinellia Tuber	*Rhizoma Pinelliae, Rhizoma Pinelliae praeparatum*	3 (2%)	3-9	9.77 ± 2.19
61	白豆蔻	Round Cardamom Fruit/ Java Amomum Fruit	*Fructus Amomi Rotundus*	3 (2%)	3-6	12 ± 6.83

62	生牡蛎	Oyster Shell	*Concha Ostreae*	3 (2%)	9-30	27 ± 7.58
63	何首乌	Tuber Fleeceflower Root	*Radix Polygoni Multiflori,Radix Polygoni Multiflori Praeparata Cum Succo Glycines Sotae*	3 (2%)	6-12	16.1 ± 5.77
64	桃仁	Peach Seed	*Semen Persicae*	3 (2%)	4.5-9	6 ± 0.00
65	乌贼骨	Cuttlefish Bone	*Endoconcha Sepiae*	3 (2%)	5-9	16.7 ± 5.26
66	黄柏	Amur Corktree Bark	*Cortex Phellodendri Chinensis*	3 (2%)	3-12	11.3 ± 2.05
67	淫羊藿	Epimedium Herb	*Herba Epimedii*	3 (2%)	3-9	15.3 ± 3.43
68	藕节/炭	Lotus Rhizome Node	*Nodus Nelumbinis Rhizomatis*	3 (2%)	9-15	11.3 ± 2.58
69	丹皮/牡丹皮	Tree Peony Bark	*Moutan Cortex*	3 (2%)	6-9	6.7 ± 1.43
70	三七	San Chi	*Radix Et Rhizoma Notoginseng*	3 (2%)	1-3	3.67 ± 1.77
71	肉苁蓉/淡大芸	Desertliving Cistanche	*Herba Cistanches*	3 (2%)	6-9	12 ± 0.00
72	棕榈炭/棕榈	Fortune Windmillpalm Petiole	*Petiolus Trachycarpi*	2 (1%)	3-9	14.8 ± 6.53
73	木香	Costustoot	*Radix Aucklandiae*	2 (1%)	1.5-6	6.86 ± 2.65
74	乌梅	Dark Plum Fruit	*Fructus Mume*	2 (1%)	6-12	10 ± 0.00
75	藿香	Wrinkled Gianthyssop Herb	*Herha Agastaches.*	2 (1%)	6-10	12.5 ± 3.44
76	红花	Safflower	*Flos Carthami*	2 (1%)	3-9	3 ± 0.00
77	牡丹	Tree Peony	*Cortex Moutan*	2 (1%)	6-12	-
78	羌活	Incised Notopterygium Rhizome/ Forbes Notopterygium	*Rhizoma Et Radix Notopterygii*	2 (1%)	3-9	6 ± 0.00

		Rhizome				
79	知母	Common Anemarrhena Rhizome	*Rhizoma Anemarrhenae*	2 (1%)	6-12	13.3 ± 3.33
80	狗脊	East Asian Tree Fern Rhizome	*Rhizoma Cibotii*	2 (1%)	6-12	15.7 ± 1.89
81	莲子	Lotus Seed	*Semen Nelumbinis*	2 (1%)	6-15	11.3 ± 2.03
82	墨旱莲	Yerbadetajo Herb	*Herba Ecliptae*	2 (1%)	6-12	15 ± 0.00
83	生龙骨	Drgon's Bones	*Os Draconis*	2 (1%)	11-18	30 ± 0.00
84	茯神	Indian Bread with Pine/ Tuckahoe with pine	*Poriacocos(schw.)Wolf*	2 (1%)	9-15	11.7 ± 2.27
85	茵陈	Capillary Wormwood Herb	*Herba Artemisiae Scopariae*	2 (1%)	6-15	22.5 ± 6.25
86	干姜/炭	Dried Ginger	*Rhizoma Zingiberis*	2 (1%)	3-9	3 ± 0.00
87	生山楂	Chinese Hawthorn Fruit	*Fructus Crataegi*	2 (1%)	9-12	25 ± 0.00
88	大青叶	Indigowoad Leaf	*Folium Isatidis*	1 (1%)	9-15	-
89	大枣	Chinese Date	*Fructus Jujubae*	1 (1%)	6-15	10
90	石菖蒲	Grassleaf Sweelflag Rhizome	*Rhizoma Acori Tatarinowii*	1 (1%)	3-9	9
91	龙眼肉	Dried Longan Prlp	*Arillus Longan*	1 (1%)	9-15	15
92	玄参	Figwort Root	*Radix Scrophulariae*	1 (1%)	9-15	-
93	地骨皮	Chinese Wolfberry Root Bark	*Cortex Lycii*	1 (1%)	9-15	-
94	合欢皮	Silktree Albizzia Bark	*Cortex Albiziae*	1 (1%)	6-12	15

95	炒麦芽	Malt	*Fructus Gordei Germinatus*	1 (1%)	9-15	13
96	远志	Thinleaf Milkwort Root-bark	*Radix Polygalae*	1 (1%)	3-9	10
97	苍术	Swordlike Atractylodes Rhizome / Chinese Atractylodes Rhizome	*Rhizoma Atractylodis*	1 (1%)	3-9	-
98	芡实	Gordon Enryale Seed	*Semen Euryales*	1 (1%)	9-15	9
99	芦根	Reed Rhizome	*Rhizoma Phragmitis*	1 (1%)	15-30	-
100	忍冬藤	Japanese Honeysuckle Stem	*Caulis Lonicerae Japonicae*	1 (1%)	9-30	-
101	枇杷叶	Loquat Leaf	*Folium Eriobotryae*	1 (1%)	6-9	10
102	侧柏/炭	Chinese Arborvitae Twig	*Cacumen Platycladi*	1 (1%)	6-12	15
103	金银花	Honeysuckle Flower	*Flos Lonicerae Japonicae*	2 (1%)	6-15	10
104	鱼腥草	Heartleaf Houttuynia Herb	*Herba Houttuyniae*	1 (1%)	15-25	-
105	泽泻	Oriental Waterplantain Rhizome	*Rhizoma Alismatis*	1 (1%)	6-9	10
106	珍珠母	Nacre	*Concha Margaritifera*	1 (1%)	10-25	30
107	枳壳	Bittet Orange	*Fructus Aurantii*	1 (1%)	3-9	9
108	厚朴	Officinal Magnolia Bark	*Cortex Magnoliae Officinalis*	1 (1%)	3-9	-
109	钩藤	Gambir Plant	*Ramulus Uncariae Cum Uncis*	1 (1%)	3-12	15
110	桑叶	Mulberry Leaf	*Folium Mori*	1 (1%)	5-9	18

111	桑螵蛸	Mantis Egg-case	*Ootheca Mantidis*	1 (1%)	5-9	-
112	黄精	Manyflower Solomonseal Rhizome / Siberian Solomonseal Rhizome / King Solomonseal Rhizome	*Rhizoma Polygonati*	1 (1%)	9-15	11
113	贯众	Cyrtomium Rhizome	*Rhizoma Cyrtomii*	1 (1%)	5-10	20
114	苏叶/紫苏槿叶	Perilla Leaf	*Folium Perillae*	1 (1%)	5-9	7.5
115	紫草	Redroot Gromwell Root	*Radix Arnebiae*	1 (1%)	5-9	15
116	蒲公英	Mongolian Dandelion Herb	*Herba Taraxaci*	1 (1%)	9-15	-
117	椿根皮	Tree-of-heaven Ailanthus Bark	*Cortex Ailanthi*	1 (1%)	6-9	10
118	覆盆子	Palmleaf Raspberry Fruit	*Fructus Rubi*	1 (1%)	6-12	16
119	沙参	Coastal Glehnia Root (North) Ladybell Root (South)	*Radix Glehniae* *Radix Adenophorae*	1 (1%)	10-15	15
120	败酱草	Dahurian Patrinia Herb / Whiteflower Patrinia Herb	*Herba Patriniae*	1 (1%)	3-9	-
121	茜草	India Madder Root	*Radix Rubiac Cordifoliae*	1 (1%)	10-15	-
122	胡麻仁	Hemp Fruit	*Fructus Cannabis*	1 (1%)	9-15	12
123	石莲子	Seed of Whiteflower Cacalia	*Semen Caesalpiniae Minacis*	1 (1%)	30-50	10
124	佛手	Finger Citron	*Fructus Citri Sarcodactylis*	1 (1%)	3-9	10

125	石斛	Dendrobium	*Herba Dendrobii Nobilis*	1 (1%)	6-12	10
126	紫河车	Human Placenta	*Placenta Hominis*	1 (1%)	2-3	3
127	金樱子	Cherokee Rose Fruit	*Fructus Rosae Laevigatae*	1 (1%)	6-12	15
128	柏子仁	Platycladi Seed	*Semen Platycladi*	1 (1%)	3-9	15
129	白及(白芨)	Tuber of Hyacinth Bletilla	*Rhizoma Platantherae Chloranthae*	1 (1%)	6-15	20
130	桑椹	Mulberry Fruit	*Fructus Mori*	1 (1%)	9-15	15

** Frequency time is referred to number of literatue of each formula/total amount of literatures *100*

# Recommended dose is referred to the recorded dose of each Chinese medicine in "ChinesePharmacopiea"

※Daily mean dose is referred to the dose of each Chinese medicine in all published literatures.
